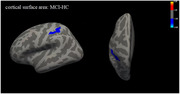# Cortical Structure of left superior parietal cortex is associated with cognition and dual tasking: a cross‐sectional study of mild cognitive impairment

**DOI:** 10.1002/alz.090234

**Published:** 2025-01-03

**Authors:** Siyun Zhang

**Affiliations:** ^1^ First Affiliated Hospital of Sun Yat‐sen University, Guangzhou, Guangdong China

## Abstract

**Background:**

Individuals with mild cognitive impairment (MCI) commonly exhibit alterations in cortical structures accompanied by a mild decline in cognitive function. While the dual‐task paradigm is utilized for MCI detection, its cortical mechanism remains incompletely understood. This study seeks to explore cortical structural changes and cognitive decline using the dual‐task paradigm in individuals with MCI.

**Method:**

Thirty participants with MCI and thirty healthy controls (HC) were recruited. Magnetic resonance imaging (MRI), cognitive assessments and dual‐task Timed Up and Go (TUG) test were performed. Cortical morphological parameters of cerebral cortical thickness and surface area were computed using Freesurfer software. Correlations analyses were then performed to assessment the relationship between cognitive, motor performance and morphological measures.

**Result:**

The execution time of the dual‐task TUG significantly increased in MCI group, and was negatively correlate with the WAIS Digit Span Forward and Backwards test, total Montreal cognitive assessment (MoCA) score, and its delayed recall subdomain. At the whole brain level, the surface area in left superior parietal cortex (SPC) was significantly reduced in MCI group. No between‐group significant variation was found in cortical thickness was observed. The surface area of the left SPC was positively correlated with total score of the MoCA and its delayed recall subdomain. In the HC group, the cortical thickness was positively correlated with total score and its attention subdomain. Negative correlation between cortical thickness and dual‐task TUG test was also observed.

**Conclusion:**

Individuals with MCI showed decline in attention, short‐term memory, inhibitory control ability and dual‐task performance. The observed structural alterations in the left SPC suggested potential distinctions in this region, which may be associated with cognitive processes and the cognitive demands related to locomotion. This study provides additional evidence supporting the role of the left superior parietal cortex in cognitive and locomotor function in individuals with MCI.